# Foamy Viruses, Bet, and APOBEC3 Restriction

**DOI:** 10.3390/v13030504

**Published:** 2021-03-18

**Authors:** Ananda Ayyappan Jaguva Vasudevan, Daniel Becker, Tom Luedde, Holger Gohlke, Carsten Münk

**Affiliations:** 1 Clinic for Gastroenterology, Hepatology and Infectiology, Medical Faculty, Heinrich Heine University Düsseldorf, 40225 Düsseldorf, Germany; tom.luedde@med.uni-duesseldorf.de; 2 Institute for Pharmaceutical and Medicinal Chemistry, Heinrich Heine University Düsseldorf, 40225 Düsseldorf, Germany; d.becker@uni-duesseldorf.de (D.B.); gohlke@uni-duesseldorf.de (H.G.); 3 John von Neumann Institute for Computing (NIC), Jülich Supercomputing Centre & Institute of Biological Information Processing (IBI-7: Structural Biochemistry), Forschungszentrum Jülich GmbH, 52425 Jülich, Germany

**Keywords:** foamy virus, retrovirus, Bet, APOBEC3, cytidine deaminase, restriction factors, mutation, viral restriction, viral antagonist

## Abstract

Non-human primates (NHP) are an important source of viruses that can spillover to humans and, after adaptation, spread through the host population. Whereas HIV-1 and HTLV-1 emerged as retroviral pathogens in humans, a unique class of retroviruses called foamy viruses (FV) with zoonotic potential are occasionally detected in bushmeat hunters or zookeepers. Various FVs are endemic in numerous mammalian natural hosts, such as primates, felines, bovines, and equines, and other animals, but not in humans. They are apathogenic, and significant differences exist between the viral life cycles of FV and other retroviruses. Importantly, FVs replicate in the presence of many well-defined retroviral restriction factors such as TRIM5α, BST2 (Tetherin), MX2, and APOBEC3 (A3). While the interaction of A3s with HIV-1 is well studied, the escape mechanisms of FVs from restriction by A3 is much less explored. Here we review the current knowledge of FV biology, host restriction factors, and FV–host interactions with an emphasis on the consequences of FV regulatory protein Bet binding to A3s and outline crucial open questions for future studies.

## 1. Foamy Viruses

The first description of a foamy virus (FV) was reported in 1954 [1]. It was found as a contaminant with an atypical cytopathic effect (CPE), eliciting the formation of multinucleated and vacuolated giant cells in primary kidney cell cultures from Old World monkeys of the Macacaceae family. The name FV or spumaretrovirus was derived from the foam-like appearance of syncytia in the infected monolayer cell cultures. FVs were classified as retroviruses after the detection of the FV reverse transcription (RT) enzyme. The first isolation of the “foamy viral agent” occurred in 1955 [2]. In 1971, a viral agent with FV-like characteristics was identified from lymphoblastoid cells in cultures of a nasopharyngeal carcinoma (NPC) from a Kenyan patient [3]. The origin of this human foamy virus (HFV) was discussed until 1994, when HFV was cloned and sequenced [4]. The 86 to 95% amino acid identity between simian foamy virus from chimpanzee (SFVcpz) and HFV suggested that HFV is likely a variant of SFVcpz and not a unique isolate [4]. Sequence comparisons between the original HFV isolate and SFV from four distinct subspecies of chimpanzee demonstrate that it is most closely related to FV from *Pan troglodytes schweinfurthii*, whose natural habitat includes Kenya. Since the original HFV isolate came from a person who might have had contact with chimpanzees in Kenya, the virus was probably acquired as a zoonotic infection (transmission from animals to humans). For detailed reviews on foamy virus epidemiology and zoonotic infections see [5,6,7]. HFV has now been renamed as the prototype foamy virus (PFV), although it is debated whether the “real” origin of the virus isolate derived from in vivo cross-species transmission from chimpanzee or from a cell culture contamination [5]. Evidence suggests that diverse SFVs are transmitted from primates to humans, but not between humans [5,8,9,10,11,12]. Notably, African green monkeys and apes have a higher prevalence for SFV, and then for simian immunodeficiency virus (SIV), and SIV-infected animals are often also positive for SFVs, indicating that co-infection of these two viruses is common in African primates [13,14,15,16,17,18].

## 2. Host-Virus Co-Speciation and Evolution

### 2.1. Simian Foamy Viruses

FV genomes display high evolutionary conservation among all the species infected, and FV genetic variability within one infected animal is very low over time (<1% variation) [19]. The phylogenetic analysis of SFV polymerase and mitochondrial cytochrome oxidase subunit II (COII) from African and Asian primates provide very similar branching order and divergence times among the two trees, supporting the co-speciation. Molecular clock calibrations have revealed an extremely low rate of SFV evolution, 1.7 × 10^−8^ base substitutions per site per year, making FV the slowest-evolving virus documented so far. These investigations moreover revealed highly congruent relationships, indicating virus-host co-evolution for at least 30–40 million years [20,21]. The various SFVs do not seem to cause any recognizable disease in their natural hosts, despite being highly cytopathic in tissue culture [5,6,22,23]. Although copying of the FV genome is highly accurate, therefore maintaining a stable genome, frequent recombination events between several circulating FV strains, as well as deletions and mutations, have been reported in wild-living chimpanzees [14,24].

SFVs are highly prevalent. In captive primate populations, infection rates ranging from 70% to 100% are reported in adult animals [5,6,23], and similarly high numbers of SFVcpz infections in wild-living chimpanzees across equatorial Africa were documented [14]. High FV prevalence was also reported in the Asian macaques (*Macaca fascicularis*) population and Wild red colobus monkeys in Tai national park, West Africa [11,25] as well as in New World primates in Central and South America [26]. A recent study identified a diversity of SFV strains in free-ranging rhesus macaques in Bangladesh [27]. Adult chimpanzees had significantly higher infection rates (13 out of 13 animals) than infants and juveniles, suggesting horizontal transmission [14]. Hunters, poachers, zookeepers, temple workers, and villagers who are occupationally exposed to non-human primates (NHP) can also become infected with SFV [28,29,30,31] (documented in [5]). Ongoing zoonotic transmissions are reported from zoos or primate centers in Gabon and in China [32,33,34], and also confirmed in hunters in Gabon, where severe bites from gorillas were causative [34]. However, humans are dead-end hosts of primate foamy viruses, and no human foamy virus has evolved so far [10,35,36].

### 2.2. Non-Simian Foamy Viruses

FVs are also prevalent in non-simian hosts such as cats [37,38], pumas [39], cattle [40,41,42], horses [43], certain bats (*Rhinolophus affinis*) [44], and likely more species (reviewed in [15,45,46,47]). Apparently, these FVs are non-pathogenic in their hosts, and the infection prevalence is recorded as about 7–45% in cattle and 30–100% in cats, mainly in adults [45,47,48,49,50]. Isolation of an equine FV and a comprehensive sero-epidemiology of infections were reported recently, with a positive rate of 25–41% [51]. These reports suggest that non-simian FV infections are highly prevalent in these animals.

### 2.3. Endogenous Foamy Viruses

When retroviruses get access to gametocytes, the resulting endogenous provirus can be passaged in the germline along with the host cell genome, but in most cases carries inactivating mutations or deletions [52,53]. Endogenous FVs were discovered in the genomes of the Madagascar aye-aye (*Daubentonia madagascariensis*, a strepsirrhine primate), the two-toed (*Choloepus hoffmanni*) and three-toed sloths (*Bradypus pygmaeus*) from South America, platyfish (*Xiphophorus maculatus*), and cod species [54,55,56], and FV-like insertions within the genome of the Coelacanth (*Latimeria chalumnae*) [57]. Very recently, 36 novel lineages of amphibian and fish foamy-like endogenous retroviruses were identified, and the ancient marine origin of retroviruses was suggested to be in the Ordovician period, early Palaeozoic Era, coinciding with the origin of jawed vertebrates [58]. Recent studies also identified endogenous FV sequences in genomes of reptiles, birds, and snakes [59,60,61,62]. Together, these results provide details of FV-host coevolution over a time of over 450 million years (MYA). These findings of newly identified ancient endogenous and an increasing number of exogenous FVs not only extend the age of FVs, but the finding in non-mammalian vertebrate phyla supports the concept of the great evolutionary success of this retroviral subfamily [63].

## 3. Molecular Biology of FV

Foamy viruses have complex RNA genomes (ranging from 10.5 kbp Feline FV [64] to 13 kbp SFVcpz [4]), which encode Tas (or bel-1) (a nuclear transcriptional transactivator) and Bet (an auxiliary protein) in addition to Gag, Pol, and Env. Of note, the Bet protein of FV is not related to “bromodomain and extra-terminal (BET) proteins”, which are cellular histone acetylation readers.

FVs display several peculiar features among retroviruses. FV Gag is translated as a precursor protein only as Gag and not also as a Gag-Pol fusion protein [65,66,67]. FV Gag lacks characteristic domains such as membrane-binding domains, the major homology regions (MHR), and the hallmark Cys-His motifs. Rather, they possess numerous specific domains, such as the essential Gag-Env interaction domain and the Gly and Arg rich boxes (GR) regions, reviewed in [68]. Moreover, FV Gag undergoes limited maturation by the FV protease. This processing of Gag (PFV pr71 Gag) does not yield matrix, capsid, and nucleocapsid (so FVs have an “immature” appearance, despite being highly infectious), but only removes a small 3 kDa C-terminal peptide resulting in p68 Gag [69,70], which is essential for FV infectivity [71,72].

**Figure 1:**
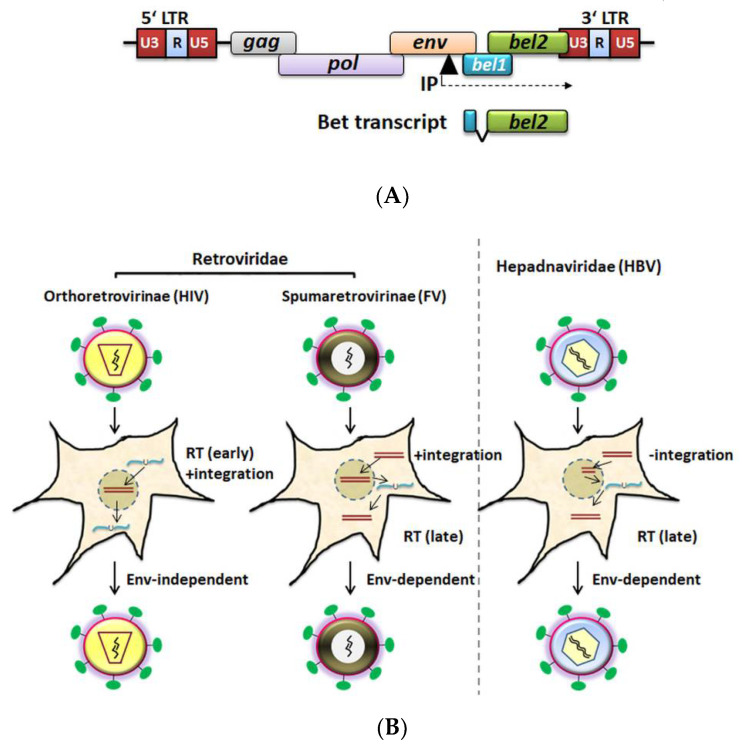
Genome organization of foamy viruses (FV) and schematic representation of replication strategies of animal viruses using reverse transcription (RT). (**A**) FV possesses *gag, pol,* and *env* genes. Additional regulatory and accessory *bel1* and *bel2* genes are localized between *env* and the 3′LTR. The Bet protein is a product of a spliced transcript and consists of Bel1 (also known as Tas) and Bel2 (alternatively called ORF2) parts. *Bel1* and *bel2* transcripts are originating from the internal promoter (IP) element, which is indicated as a black triangle, and the direction of transcription is marked with a dashed arrow (figure modified from [89]). (**B**) Replication cycle: Orthoretroviruses (RNA genome in the virion) such as HIV-1 replicate through a dsDNA intermediate and require integration of viral genome into host genome for propagation. Hepadnaviruses (dsDNA in the virion) instead do not integrate their genome but require an RNA intermediate (late RT) for their replication. FVs integrate their genome (DNA) into the host chromosomal DNA like orthoretroviruses, but undergo late reverse transcription like hepadnaviruses. It is controversially discussed in the literature that FV virus particles encapsidate genomic viral RNA or already reverse-transcribed DNA, and the exact copy numbers of both viral genomic RNA and DNA in “cell-free” FV particles remains to be determined in vivo [14,67,82,86,87,90]. The release of FV viral particles depends on the FV glycoprotein [91] as in hepadnaviruses, while budding of orthoretroviral particles is Env-independent. RT, reverse transcriptase, RNA and DNA molecules are denoted by blue lines with U and brown lines respectively. Arrows in the figure indicate the path of the viral replication event. (Figure adapted from [63] and taken from one author’s thesis [92]). Hepadnaviruses are depicted here to illustrate different viral replication strategies of animal viruses with a reverse transcription step.

Unlike Pol proteins from orthoretrovirinae, FV Pol is synthesized independently of Gag (from a singly spliced pol mRNA), and encapsidation of Pol is thought to be mediated by viral RNA bridging Gag and Pol molecules [73,74,75,76]. The fidelity of PFV PR-RT with respect to base substitutions was suggested to be similar to that of HIV-1 RT, although it can generate more insertions and deletions [77]. In comparison with the DNA polymerase processivity of HIV-RT, the processivity of PFV PR-RT is higher; therefore, having a few Pol molecules in the viral particles might be sufficient for productive infection [77,78,79,80]. In contrast to the orthoretroviruses, FV reverse transcription takes place to some extent (5–10%) late in the infection cycle (before the virus leaves the cell) [67,81,82]. During virus assembly, the pregenomic ssRNA (as a dimer) is incorporated and can be reverse transcribed to dsDNA before virus release (Figure 1). While it is generally accepted that viral genomic DNA contributes to productive infection during the spreading of FVs in cultures, both viral genomic RNA- and DNA-containing particles are found in the supernatant of FV-infected cells. Furthermore, studies conducted with reverse transcriptase inhibitor AZT (3′-azido-3′deoxythymidine) indicate that reverse transcription is mostly complete prior to an extracellular virus infecting new cells [67,76,81,83,84,85,86]. A few studies supported the existence of an early RT step during foamy virus infection upon entry, which is thought to be relevant at low multiplicities of infection [82,87]. The biological relevance of virions with DNA or RNA genomes is controversially discussed, and the precise proportion of these needs to be determined, especially in vivo [14,67,81,82,86,87,88].

FVs are unable to establish a productive infection in G1/S growth-arrested or nondividing cells [93], requiring mitosis for proviral integration and gene expression [93,94,95,96]. *In vitro*, FVs have the capacity to infect most cell types of vertebrate origin from fish to humans [45,97,98]. Heparan sulfate proteoglycans are suggested as an attachment factor for FV entry [99,100]. Glycoprotein-dependent FV entry into the host cells is achieved by pH-dependent endocytosis and, alternatively, the release of naked FV capsids (cores) into the cytoplasm after fusion with the plasma membrane. Viral cores are then shuttled along microtubules and accumulate at the microtubule organizing center (MTOC). Uncoating of capsids assisted by the host and viral proteases occurs during mitosis, and ultimately, the preintegration complex (PIC) gets access to the chromatin (via Gag tethering) for the viral genome integration [101,102,103,104], reviewed in [105,106]. Whereas studies on FV cellular tropism in vivo are very limited, some reports indicated that FV DNA was detected in CD4+ and CD8+ lymphocytes, monocytes, and B-cells in humans, African green monkeys (AGM), chimpanzee, gorilla, and cattle [10,107,108,109,110]. One study suggested that the niche of in vivo FV replication in primates is limited to the differentiated superficial epithelial cells of the oral mucosa, a short-lived reservoir, resulting in nonpathogenic infections [111].

## 4. Innate Immune Sensing of Foamy Viruses

Like other viruses, FVs not only have to exploit various host machinery for their productive replication, but they have also to escape or counteract host antiviral responses, such as innate immune sensing and inhibition by cellular restriction factors. This area of research is particularly interesting because FV infections are apparently apathogenic in the hosts, but in vitro, FV infection triggers cytopathic effects and ultimately leads to cell death [22]. However, recent case–control studies among Cameroonian hunters infected with gorilla SFV identified an association of T-cell differentiation, monocyte activation, and hematological abnormalities with SFV infection [112,113]. These and a similar study that characterized an association of FFV with chronic kidney disease in cats [114] suggest that more research is needed to explore in vivo pathological changes by FVs.

Innate sensing of FV is not well characterized. Early studies suggested a lack of type I interferon (IFN-I) induction by simian and human FV infections in different cell lines [115,116,117]. However, a later study demonstrated that FVs are sensed by human hematopoietic cells such as PBMCs and pDCs potently inducing the production of IFN-I and associated IFN-stimulated genes (ISG), like MX1 [118]. This study further highlighted that RNA but not DNA is responsible for the trigger, and TLR7 was identified as the main sensor. In cell culture, treatment with IFNs led to viral inhibition [116,117,119,120,121,122,123]. Our own results also supported the inhibition of various FVs by IFN-β, but not by the ISG product MX2 [124]. Additionally, a new study suggested that pharmacological inhibitors of the IFN-I response enhance the replication of primary gorilla SFVs [125]. Very recently, using a myeloid cell model, the innate sensing pathways involved in FV infection were described [88]. Efficient ISG induction was demonstrated by sensing of full-length PFV genomes but not of minimal vectors in the cytoplasm. Moreover, this study suggested that viral DNA but not RNA acts as the key stimulator since this innate response was mainly dependent on cellular cGAS and STING, and unaffected by RT inhibition during entry [88]. Foamy viral escape from sensing may be mediated in PFV infections by Gag-induced endosomal autophagy that facilitates the clearance of stress granules to repress an IFN-I response of the infected cell [126]. In addition, FV encoded micro-RNAs (miRNAs) as shown in BFV [127] and SFVs [128] could act as regulators of the innate immune response. BFV miR-BF2–5p can suppress the expression of IFN-β and NF-kB mRNAs by targeting Ankyrin Repeat Domain 17 (ANKRD17), an upstream regulator in the innate immune system and Bax-interacting factor 1 (Bif-1, official name SH3GLB1) [129].

### Foamy Virus Inhibition by Cellular Restriction Factors

Specific IFN-induced cellular gene products are able to restrict different retroviruses, including FVs [121,122,130,131,132,133,134,135,136,137,138,139,140,141,142]. Similar to other retroviruses, TRIM5α targets the core of PFV, SFV, and FFV during early post-infection events in a species-dependent manner [135,139,143]. The broad-spectrum of retroviral particle release inhibition by BST2 (tetherin) was reported to include FVs [131,132,137]. Fv1, a rodent restriction factor that inhibits murine leukemia virus (MLV), specifically, the Fv1 from *Mus caroli*, was found to inhibit FFV [138,144]. Human interferon-induced 35 kDa protein (IFP35), an interferon-induced leucine zipper protein, was reported to confer resistance to BFV and PFV infection by inhibiting Tas of BFV and PFV [142]. Furthermore, proteasomal-dependent degradation of PFV Tas by an E3 ubiquitin ligase, human p53-induced RING-H2 protein (PIRH2, official name RCHY1), was identified. PIRH2 was demonstrated to inhibit PFV replication and decreased the Tas-dependent transcriptional activation of the viral LTR and internal (IP) promoter [145]. A recent study using a screen of ISGs identified PHD finger domain protein-1 (PHF11) as an additional inhibitor of PFV [133]. Interestingly, PHF11 from humans and macaques were reported to be antiviral against multiple FVs but to be inactive against orthoretroviruses; thus, it appears to be FV-specific. PHF11 targets basal Tas expression by the IP (Figure 1A). As a consequence, Tas-dependent LTR activation is prevented, likely promoting viral latency [133]. Moreover, human/bovine N-Myc interactors (NMI) [146] and human interferon-induced transmembrane (IFITM) proteins [141] were identified to restrict PFV/BFV and FFV replication, respectively [141].

Another type of innate frontline defense against retroviruses is mediated by the members of the APOBEC3 (A3) cytidine deaminases family. Our earlier study reported the DNA editing of the FFV genome in A3-positive feline CRFK cells (non-permissive phenotype), which dramatically diminished the FFV titer [134]. Two other studies demonstrated A3-mediated (A3C, A3G, A3F from human, mA3, and cpzA3G) inhibition of PFV infectivity, correlating with encapsidation of A3 into PFV vectors due to a specific Gag-A3 interaction and cytidine deamination of the viral reverse transcripts that resulted in G-to-A hypermutation of the viral genome. Of note, both studies used PFV-based single-round replication vectors [130,136]. Using a new statistical approach, A3G-mediated G-to-A hypermutation was detected and quantified in macaques and humans who were zoonotically infected. These data suggest that human A3G but not simian A3s induce hypermutations, which are lethal to the virus, hence protecting humans from SFV transmission [27,147]. The following chapters discuss briefly A3 proteins, and then focus on Bet protein and A3s-FV interactions. The important role of the Bet protein in counteracting A3s and various Bet-A3 interactions that lead to the escape of FVs are highlighted.

## 5. APOBEC3s: Eutheria-Specific Antiviral Polynucleotide DNA Cytidine Deaminases

The apolipoprotein B mRNA-editing enzyme, catalytic polypeptide-like 3 (APOBEC3, A3) family of single-stranded (ss) DNA cytidine deaminases provides mammals with an innate immune barrier against retroviruses, retrotransposons, and other viral pathogens [148,149,150,151,152,153]. All placental mammals encode at least one A3 gene. Whereas rodents possess single A3 (mouse *A3*, *mA3* for instance) genes, humans express seven A3 proteins, and bats likely up to 18 distinct A3s from as many genes [149,154,155,156]. The A3 proteins have either one or two zinc-coordinating DNA cytidine deaminase domains (Z). Human A3A, A3C, and A3H possess one and human A3B, A3D, A3F, and A3G two Z-domains, respectively, but only one Z domain is catalytically active in each A3 [148,149,157,158].

The domain structure of A3s consists of a conserved sequence of characteristic motifs (α1-β1-β2/2′-α2-β3-α3-β4-α4-β5-α5-α6) [151,152]. At first A3G, then A3F, and other A3s were described as restriction factors because of their ability to inhibit the replication of Vif-deficient HIV-1 [150,151,153,159,160]. Based on the prevailing model of retrovirus restriction, A3s interact with viral components such as capsid, nucleocapsid, and nucleic acids to become encapsidated into nascent virions. Upon infection of target cells, the passenger A3s in the viral core deaminate the negative strand cDNA formed during reverse transcription of the viral RNA. A3s induce hypermutation (C-to-T deamination) on this single-stranded DNA, thereby editing the coding viral DNA strand (G→A), leading to the inhibition of productive viral infection. Additional modes of inhibition of retroviruses are also well established that are not dependent on deaminase activity [161,162,163]. Based on cell-type and tissue, A3 expression is constitutive or inducible (e.g., by interferons). In general, A3s are expressed widely in hematopoietic cells [164,165,166,167,168].

During infection by lentiviruses (a group of retroviruses that includes HIV-1) the antiviral activity of the host-species’ A3 is counteracted by the viral Vif protein. Vif directly binds to A3 and recruits an E3 ubiquitin ligase complex for polyubiquitination and proteasomal degradation of A3 [169,170,171,172,173]. This leads to intracellular depletion of A3s and allows the production of infectious viral particles that are mostly devoid of A3s. In addition, degradation-independent mechanisms such as Vif-mediated inhibition of A3G translation and A3G deaminase activity are also known [174,175,176,177,178]. Furthermore, retroviruses that lack a functional Vif-like gene have evolved different viral proteins and alternative countermeasures to prevent the antiviral activity of A3s. For instance, murine leukemia virus (MLV) glycoGag (a glycosylated translation product of the *gag* gene that is initiated by an upstream CUG codon (p80)), can antagonize mA3 and certain human A3s’ restriction activity [168,179,180,181,182]. GlycoGag protects MLV by providing sufficient stability to viral cores, thereby hindering mA3 access to the MLV-RT complex in target cells. GlycoGag was shown to protect the genomes of some MLV strains from mA3 deamination; however, the precise mechanism of this protection remains to be elucidated (reviewed in [162]). Along the same lines, the Bet accessory protein from several FVs was demonstrated to sequester A3s in a degradation-independent manner [134,136,183,184,185].

## 6. FV Auxiliary Protein Bet

FVs encode two non-structural proteins, Tas and Bet, which are implicated in overcoming host innate immunity. Whereas the transcriptional activator function of Tas and its role in inhibiting RNA silencing pathways have been known for some time [186], a dedicated function of Bet was not described until recently. The FV accessory protein Bet is a unique protein with no similarities to other viral or cellular proteins [187]. Presumably, Bet is highly expressed in infected primates, cats, and cows, since antibodies against Bet and Bet expression are constantly detectable and are considered to be of diagnostic value [49,108,188,189]. Bet is likely a phosphoprotein [188], but the modified residues and potential kinases are unknown. Bet is found in vast amounts in the cytoplasm of infected or transfected cells [190,191], and it may localize to the nucleus by its C-terminal NLS [192]. Bet may also be secreted via unconventional exocytosis (not dependent on ER-to-Golgi secretory pathway) and enter uninfected cells [192]. While Bet is not required for FV infection in vitro in most assays [193,194,195,196], it has been proposed to regulate viral latency [192,197,198], establishing infection and maintaining persistence [199], to be involved in resistance to viral superinfection [200], and Bet of bovine FV (BFV) was characterized as a negative regulator of BFV replication [201]. In addition, FFV Bet is essential for infectivity and appears to have a role in viral particle release [202]. Evidence for an essential function independent of inhibiting antiviral A3 proteins was obtained by in vivo infections of FFV that expressed the feline immunodeficiency virus (FIV) Vif protein instead of Bet [203]. Such chimeric viruses replicated in feline A3-expressing cells, but were attenuated in vivo in cats.

## 7. Structure and Function Prediction of Bet

**Figure 2:**
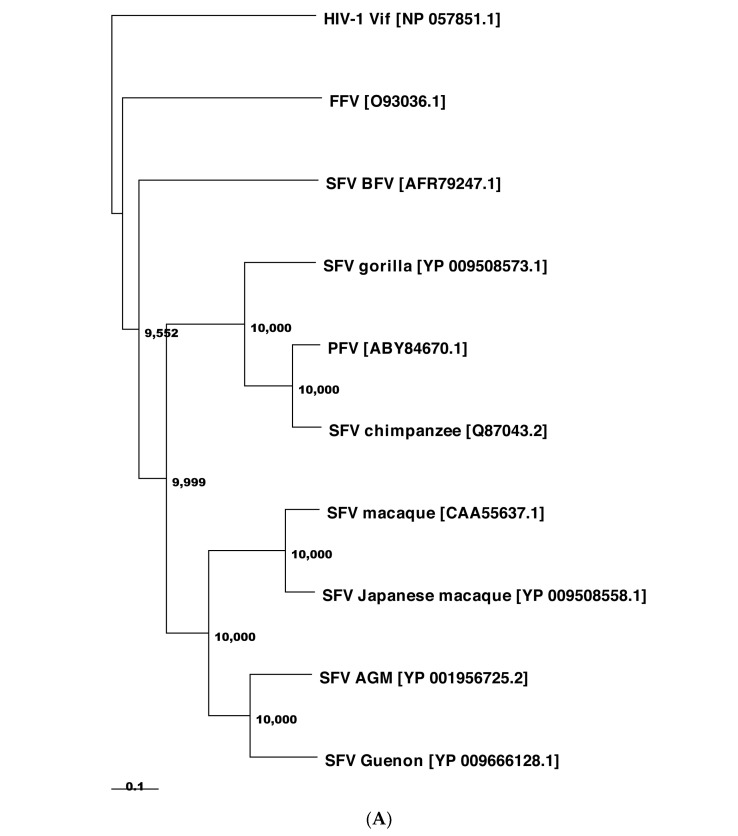

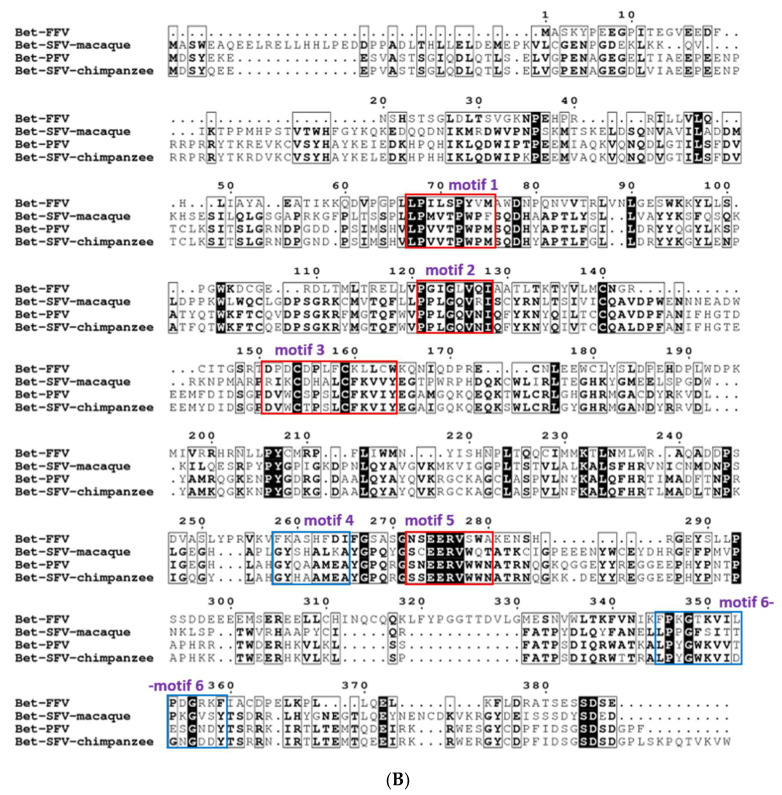
The diversity of Bet proteins. (**A**) Phylogeny of FV Bet amino acid sequences. A neighbor-joining tree based on Bet protein sequences was calculated by using the Maximum Composite Likelihood method with a bootstrap test of 10,000 replicates. Bet from BFV, FFV, SFVs (from gorilla, chimpanzee, macaque, Japanese macaque, AGM, and Guenon); HIV-1 Vif served as the out-group. Bar, 10% sequence variation. Accession numbers are provided for each sequence in the bracket. Note that we could not include Bet from other exogenous and endogenous FVs due to the lack of sequence description. (**B**) Multiple sequence alignment of Bet from FFV, PFV, and SFVs. Amino acid sequence identity and similarity among these Bet proteins are 6.7% and 64%, respectively. Additional sequence alignment leaving FFV Bet exhibited 32% identity and 79% similarity of primate FV Bet sequence (alignment not shown). Sequence alignment was performed by Clustal Omega (http://www.ebi.ac.uk/Tools/msa/clustalo/, accessed on 6 December 2020). The alignment file was then submitted to ESPript 3.0 [204] (espript.ibcp.fr) to calculate the similarity and identity of residues between these proteins and to represent the multiple sequence alignment. Six conserved motifs in Bet sequences are marked by boxes, from which four red-colored motifs were experimentally studied due to high sequence conservation, as reported before [89].

The extent of protein sequence homology of Bet is low among FVs [43,48,89]. The Bet proteins from PFV, SFVs, and FFV are rather diverse and share only 6.7% of amino acid identity and 64% of similarity. PFV and SFV Bet proteins from chimpanzee and macaque have a higher identity and similarity, i.e., 32% and 79%, respectively (Figure 2).

Despite recent advances in *ab initio* protein structure prediction [205], template-based structure modeling is still considered to yield high-quality structural models if appropriate template structures can be identified [206]. However, using the full-length sequences of the PFV, SFV-macaque, SFV-chimpanzee, and FFV Bet proteins, none of the template-based methods TopModel [206,207,208,209], I-TASSER [210,211,212], Maestro Homology Modelling [213], or SWISS-MODEL [214,215] yielded structural models of sufficient quality. This is probably due to the lack of templates with a high enough sequence identity; e.g., the best template found by I-TASSER for the SFV-chimpanzee Bet protein was 2OCW_A, with an identity of 12.4%. Predicting structural models *ab initio* based on the full sequences with ROSETTA3 [216] or the ROBETTA webserver [217] did not yield results of sufficient quality either. Finally, modeling the full-length sequences with constraints from coevolutionary information is not possible either because the GREMLIN webserver [218,219,220] or Blast [221] searches did not identify enough homologous sequences for reliably detecting residues that coevolved.

**Figure 3:**
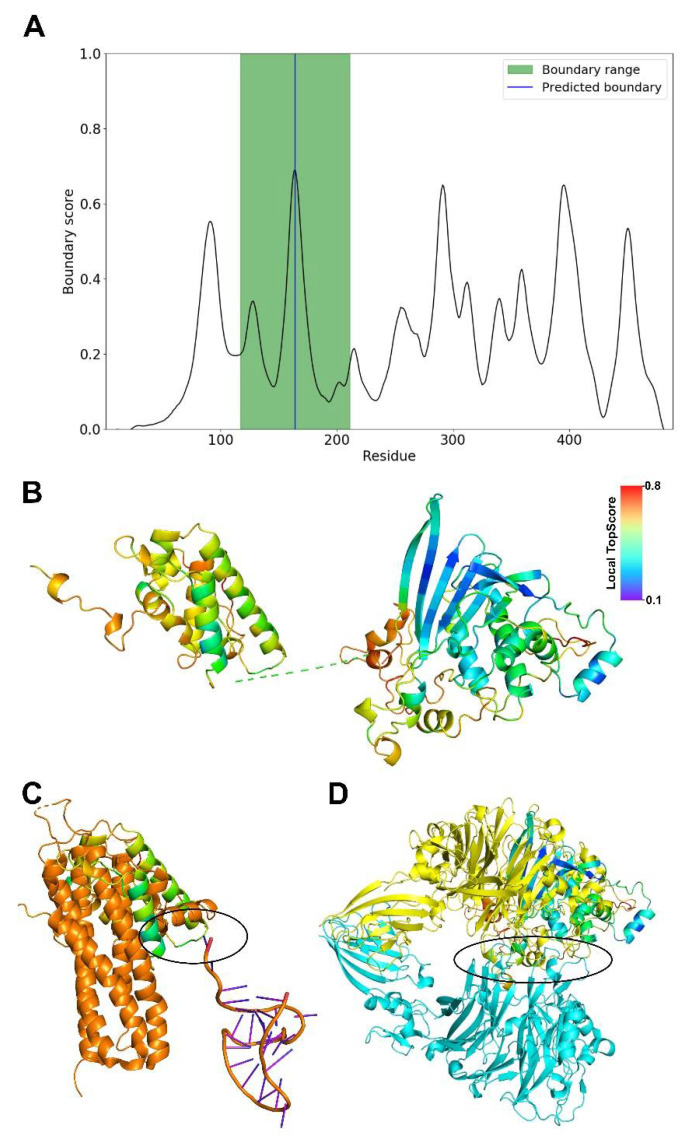
Molecular model of SFV-chimpanzee Bet protein domains. (**A**) Domain boundaries predicted by TopDomain [222]. The blue line indicates the split of the sequence into two domains (between residues 164 and 165), which were subsequently modeled separately. (**B**) Domains are shown in cartoon representation. The coloring indicates the quality assessment of the models on a per-residue level by TopScore [223]. Blue: TopScore = 0.1 (high structural quality), red: TopScore = 0.8 (low structural quality). The dotted line represents the connection between domain 1 (left, residues 1–164) and domain 2 (right, residues 165–490). Structural models were generated with ROBETTA [217]. The mutual spatial arrangement of both domains is unknown. (**C**) Overlay of the C-terminal domain and PDB ID 5M0I_C. The interaction region with mRNA is marked in black. The C-terminal domain is colored as in panel B, 5M0I is colored in orange. (**D**) Overlay of the N-terminal domain and PDB ID 6PQ8_A. The interaction region with chain B of 6PQ8 is marked in black. The C-terminal domain is colored as in panel B, 6PQ8 chain A is colored in yellow, 6PQ8 chain B is colored in blue.

Therefore, we decided to predict domain boundaries within the sequence of the SFV-chimpanzee Bet protein sequence with TopDomain [222] (Figure 3A) and subsequently modeled the two domains using the ROBETTA webserver. For each domain, ROBETTA generated five models. The best model (Figure 3B) was then chosen based on the lowest C_α_ atom RMSD between the generated models and the corresponding *ab initio* modeled domain of PFV Bet. This consensus approach was also validated by TopScore [223] in that domains with the lowest RMSD also have the lowest TopScore compared to the other four models. For the N-terminal domain, the overall TopScore is 0.47 and for the C-terminal one 0.45, indicating a moderate quality of the models. In the N-terminal domain, the helical part of the structure has low local TopScores, indicating good quality, but the N-terminal loops high local TopScores, indicating low quality. In the C-terminal domain, the β-strands have low local TopScores, indicating good quality, but helices and loops from residues 358–403 have high local TopScores, indicating low quality.

For the N-terminal domain, the DALI web server [224] identifies the ATP-binding subunit of the KdpFABC complex (6HRA_B) as the closest structural homolog (Z-score = 3.4, identity = 3%, RMSD = 4.5 Å), but the overlaying part consists of transmembrane helices. The second best hit (6IKN_A) is also a membrane protein, which we deem inapt for a viral Bet protein. The third best hit (6W2W_A) is a synthetical protein. The fourth best hit (5M0I_C, Z-score = 2.9, identity = 13%, RMSD = 4.3 Å) is part of the *ASH1* mRNA transportation complex [225] (Figure 3C), from which a potential nucleic acid interaction region can be derived. For the C-terminal domain, the first five hits (4QL0_A, 7BTX_L, 4C00_A, 3CSL_A, 3KVN_X; Z-scores 4.8 to 4.4) from the DALI webserver are structures with β-sheet barrels, although the predicted domain structure does not form a barrel. The sixth hit is 6QP8_A (Z-score = 4.4, identity = 3%, RMSD = 4.5 Å) (Figure 3D), which is part of a signaling cascade and engages in protein–protein interactions [226], from which a potential interaction interface can be derived. The results suggest that the N-terminal domain may be involved in DNA or RNA binding and the C-terminal one in the recruitment of another protein.

Due to the lack of coevolutionary information for residue pairs between the domains, as tested using HDock [227], and the uncertainty of protein–protein docking of unbound structures [228], we refrained from modeling the protein’s quaternary structure based on the two domains.

As for the other FV Bet proteins, we expect that the PFV Bet and SFV-macaque Bet proteins have structures similar to the SFV-chimpanzee Bet protein due to sequence identities of 86.2% and 33.14%, respectively (Figure 2). The FFV Bet protein is sequentially more distant, as indicated that it is not part of the same Pfam [229] entry (PF03274) as the other three Bet proteins.

## 8. FV Bet-APOBEC3 Interaction: A Distinct Retroviral Strategy to Protect Viral Genomes without Inducing Degradation of APOBEC3s

Using FFV Bet and cat CRFK cells, our study in 2005 pioneered the role of Bet in counteracting feline A3 (feA3)-mediated retroviral inhibition [134]. FFV permissive CRFK cells displayed a nonpermissive phenotype when infected with Bet-defective FFV [202], and further investigations uncovered A3-mediated genome editing of FFVΔBet [134]. Of note, in contrast to lentiviruses, the most distinguishing feature is that the deamination activity on viral substrate DNA already occurs in FFV-producing cells, so that edited-proviral DNA genomes are present in released virions. Interestingly, the absence of Bet from FFV genomes resulted in strongly decreased FFV titers, diminished particle release, and attenuated Gag processing, in addition to pronounced genome editing by A3 [134]. Furthermore, FFV Bet binding to feA3, but not human A3G, was observed, indicating a species-specific interaction of these proteins. Whereas feA3 was encapsidated into the virions, Bet was not. This study and follow-up studies further confirmed the specific interaction of FFV Bet with feA3s, which results in A3 sequestration in cells, probably as an “immobile complex”, without triggering proteolytic degradation of feline A3s [89,134,158,203,230]. Like FIV Vif, which binds to the different feA3s and promotes their degradation, FFV Bet interacts with all feA3s, independent of whether they restrict the FFV or not [230]. In contrast to Bet-feA3 binding, only some feA3s that contain a Z2 domain (feA3Z2 and feA3Z2-Z3) bind to FFV Gag. This Gag–A3 interaction is found to be crucial for feA3 incorporation into progeny virions and subsequently for restriction [230,231].

Computational analysis of Bet sequences from BFV, EFV, SFV, FFV, PFV, and SloEFV identified six conserved motifs in the Bel2 domain of Bet [89]. The entire FFV Bel2 domain, but not the N-terminal Tas region, is essential for feA3 binding and inactivation [89]. Although the Bel1/Tas motif seems dispensable, intriguingly, it increases the stability of Bet. Hence, both Tas and Bel2 regions are needed for efficient counteraction of feA3 [89]. In addition, this study indicated that the Bel1 region and C-terminal 22 residues of the FFV bel2/bet ORF can be exchanged by related FVs, such as PFV, even though PFV Bet does not bind and counteract feA3Z2. A mechanism for FFV Bet activity has not been identified, but an interaction of FFV Bet and feline A3s was demonstrated to be necessary [89,230]. A recent investigation using a cat model and a chimeric FFV system suggests that FIV Vif can replace Bet to counteract feA3 in cell lines and animals. This replacement yielded replication-competent chimeric FFVs that replicated and spread in cell culture but were attenuated in vivo [203]. These findings underscore the essential requirement of Bet and possibly additional functions other than A3 antagonism in vivo for productive FFV infection.

**Figure 4:**
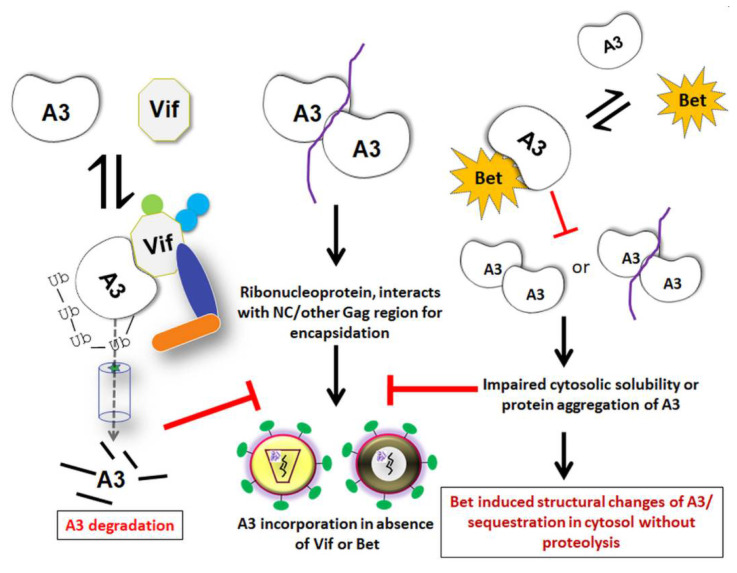
Different modes of suppression of A3 viral incorporation by lentiviral Vif and FV Bet. Schematic representation of APOBEC3 (A3) counteraction strategies developed by lentiviral Vif and foamy viral Bet proteins. While Vif binds and triggers proteasomal degradation of A3 proteins by recruiting an E3 ubiquitin ligase complex (left model), Bet differs from Vif in that it specifically interrupts the self-association of A3s, thereby diminishing their cytosolic solubility without inducing their degradation (right model). Either way, these retroviral accessory proteins overcome A3-mediated inhibition of viral infectivity by depleting/sequestering them away from progeny virions. For simplicity, A3-Vif and A3-Bet complex formation are depicted as two monomers interacting with each other; homodimers of Vif, A3, and Bet are not shown. Only RNA (purple line)-dependent A3 oligomers or A3-A3 dimers are illustrated to indicate for viral incorporation and as a target of Bet inhibition.

Notably, two earlier studies using the PFV-based viral vector system reported controversial data regarding the function of Bet as an A3G antagonist [130,136]. Russell et al. [136] demonstrated that human A3G and A3F proteins inhibit the infectivity of PFV due to a specific Gag–A3 interaction and induce cytidine deamination in the PFV genomes. They also demonstrated binding of PFV Bet to human A3F and A3G (and rescue of PFV), but not to mA3. Additionally, PFV Bet and Bet from AGM FV could rescue the infectivity of PFVΔBet and Vif-deficient HIV-1 in the presence of AGM A3G by blocking the packaging of A3G into HIV-1 virion particles [136]. Like FFV Bet, these Bet proteins as well did not deplete A3 levels in viral producer cells (Figure 4). In contrast to this report, Delebecque et al. [130] found that A3G and A3F can effectively restrict PFV by their deaminase function, independently of Bet. They also found that in addition to human A3G and A3F, A3G from chimpanzee, AGM, and mA3 all restricted FV replication in cell culture irrespective of Bet, whereas human A3B and A3C did not inhibit PFVΔBet. Importantly, these authors could not observe a Vif-like activity of Bet, i.e., FV Bet did not rescue HIV-1ΔVif in the presence of human A3G, but this study did not investigate Bet-A3 interaction [130].

To learn more about mechanistic aspects and functional consequences of Bet binding to A3s, our lab has conducted two independent studies involving A3C and A3G. Both have reported a direct physical interaction of Bet with A3C and A3G, independent of RNA, which traps the A3s in the cytoplasm rendering them unavailable for incorporation into progeny virions [183,184]. The Bet-dependent counteraction of A3-mediated restriction was reproduced using SIV-based reporter vectors, in addition to PFV vectors. Although PFV Bet displayed inhibitory activity against a range of simian A3s, it failed to interact with rhesus A3C [184]. Interestingly, the mapped binding site of Bet in A3C and A3G was found to be a region that is involved in A3 homodimerization or multimerization. The PFV Bet interactions with A3s thus impair protein self-association of A3s, without inducing their proteasomal degradation [183,184]. RNA-dependent oligomerization of A3s is crucial for their packaging into nascent HIV-1 virions [232], but it has not been investigated in the FV system. While Bet targets the nuclear fraction of A3C to the cytoplasm, it is unknown whether Bet drags A3C into an insoluble complex or aggregates to keep them immobile [183]. However, it was demonstrated that Bet sequesters A3G in immobile complexes and abrogates the cytosolic solubility of A3G by inhibiting A3G–A3G interaction [183]. Notably, Bet did not inhibit the catalytic activity of A3G, but a direct interaction of purified A3G and Bet proteins was demonstrated [183]. These studies indicate a similar mechanism of inhibition of A3s by Bet, acting mainly when the Bet expression is sufficiently high.

Additionally, our recent study suggested that Bet can counteract A3B without decreasing the steady-state level of A3B [185]. Intriguingly, Bet-A3B complex formation led to the shifting of nuclear A3B to the cytoplasm, therefore affecting its cellular localization. Another salient feature of Bet binding to A3B and A3G was studied by velocity sedimentation as these A3 ribonucleoproteins are found as RNA-bridged high-molecular-mass (HMM) complexes in the cell [183,185,232,233,234]. Bet prevented the HMM complex formation of A3G and A3B. Notably, Bet interaction with A3G was clearly demonstrated to be RNA-independent [183,185]. Thus, it would be interesting to understand the impact of Bet on other A3 HMM complexes such as those including A3F and A3C that are either insensitive to RNase or critically stabilized by RNA molecules [235,236,237]. Because nuclear localization of A3B is a primary cause of and essential for cancer mutations [238,239,240,241,242,243], this study provides a perspective of using Bet-like proteins to protect host genomes from A3-mutations that cause tumor progression and therapy resistance by targeting nuclear A3B.

## 9. Perspectives, Open Questions, and Challenges

Several investigations identified Bet as a countermeasure to protect viruses from A3s by blocking packaging into progeny virions, a function reminiscent of lentiviral Vif proteins. The reverse transcription complex of FV is not well described, and it is unknown how the antiviral A3 proteins get co-packaged in such complexes. Additional work focusing on A3 interactions with different FV Gag, viral genomic RNA, and DNA is needed to confirm the role of A3 in producer and target cells using both authentic foamy-viral as well as lentiviral systems.

There has been impressive progress in biochemical and structural understanding of A3 and HIV-1 Vif, and molecular studies of purified Bet proteins remain for future work. Likewise, A3 binding motifs of Bet were not comprehensively studied; while the physical interaction of Bet-A3 and Bet binding regions on A3s were demonstrated, we do not know much about the key regions of Bet involved in these interactions. Indeed, structural investigations on Bet will be important, but are complicated by its folding/stability issues (personal communication, see above, Bet structure and function prediction section). Whereas it was demonstrated that Bet impairs the cytosolic solubility of A3s and drags A3s into insoluble complexes in the cell, a direct/indirect function of Bet in shielding viral DNA during RT (late) from A3s in target cells has not been proposed. Additionally, nuclear-localized A3A, A3B, and A3C may impede Gag tethering, genome integration, and transcription of Tas/Bet or LTR-driven viral genes. These open questions and the discovery of currently unknown functions of Bet identification await future research.

## Data Availability

The molecular models can be downloaded from researchdata.hhu.de, the DOI is “http://dx.doi.org/10.25838/d5p-17”.
